# Preparation and Characterization of PEBAX-5513/AgBF_4_/BMIMBF_4_ Membranes for Olefin/Paraffin Separation

**DOI:** 10.3390/polym12071550

**Published:** 2020-07-13

**Authors:** So Young Kim, Younghyun Cho, Sang Wook Kang

**Affiliations:** 1 Department of Chemistry, Sangmyung University, Seoul 03016, Korea; rlathdud625@naver.com; 2 Department of Energy Systems Engineering, Soonchunhyang University, Asan 31538, Korea; 3 Department of Chemistry and Energy Engineering, Sangmyung University, Seoul 03016, Korea

**Keywords:** olefin, paraffin, copolymer, facilitated transport, ionic liquid

## Abstract

In this study, we investigated a poly(ether-block-amide)-5513 (PEBAX-5513)/AgBF_4_/1-butyl-3-methylimidazolium tetrafluoroborate (BMIMBF_4_) composite membrane, which is expected to have a high stabilizing effect on the Ag^+^ ions functioning as olefin carriers in the amide group. Poly(ethylene oxide) (PEO) only consists of ether regions, whereas the PEBAX-5513 copolymer contains both ether and amide regions. However, given the brittle nature of the amide, the penetration of BMIMBF_4_ remains challenging. The nanoparticles did not stabilize after their formation in the long-term test, thereby resulting in a poor performance compared to previous experiments using PEO as the polymer (selectivity 3; permeance 12.3 GPU). The properties of the functional groups in the polymers were assessed using Fourier transform infrared spectroscopy, scanning electron microscopy, and thermogravimetric analysis, which confirmed that the properties endowed during the production of the film using the ionic liquid can impact the performance.

## 1. Introduction

Olefins, otherwise known as alkenes, are hydrocarbons with double bonds, whereas paraffins are hydrocarbons containing single bonds. Paraffins are typically used as fuels, whereas olefins can be utilized as polymer monomers because they have double bonds. However, high-purity olefins are required in order to be used in polymerization [1]. Olefin/paraffin separation is an essential process because olefins are produced with the corresponding paraffins. At present, olefin/paraffin separation has primarily been achieved through cryogenic column distillation because olefin and paraffin have extremely similar characteristics [2,3]. However, cryogenic distillation is an expensive and energy-intensive process requiring the use of expensive equipment [4]. Therefore, to address this limitation, considerable research is being conducted on olefin/paraffin separation using a membrane [5,6]. Membrane separation is more advantageous than cryogenic distillation in terms of the energy consumption, eco-friendliness, and equipment. In addition, membrane separation is not only used in olefin/paraffin separation, but can also be applied to gas mixtures such as CO_2_/N_2_, CO_2_/CH_4_, O_2_/N_2_, N_2_/SF_6_, and H_2_/O_2_ [7,8,9,10,11]. However, it is difficult to simultaneously increase the selectivity and permeability in membrane separation due to the trade-off phenomenon [12,13]. In addition to the trade-off phenomenon, as a method for solving aging and plasticization, mixed matrix membranes (MMM) containing an organic inorganic material, such as zeolite carbon nanotubes, have been studied [14,15].

The concept of facilitated transportation was applied as a solution to the trade-off phenomenon [16]. Facilitated transportation refers to general dissolution and diffusion, as well as the concept of separation using a carrier. Transition metal cations can generally form double bonds and complexes [17,18]. Silver ions are representative olefin carriers that are applicable in this capacity due to their ability to reversibly form complexes with olefins [19,20]. Paraffin only permeates the membrane by dissolution and diffusion; however, the carriers in olefins simultaneously increase the selectivity and permeance.

However, the sole use of silver ions as carriers becomes a disadvantage when the ions are reduced to silver nanoparticles due to their inability to continue functioning as carriers, thus exhibiting poor performance. Consequently, several studies have been conducted to improve the long-term stability of silver ions by adding an oxidizer, stabilizer, or a similar substance to prevent performance deterioration due to the reduction of silver ions [19,21,22,23,24]. Moreover, a method involving the use of metal nanoparticles with polarized surfaces as carriers has also been studied [25,26]. Electron acceptors, such as tetracyanoquinodimethane (TCNQ), p-benzoquinone (p-BQ), and ionic liquids (ILs), are required to polarize the silver nanoparticles [27,28].

We conducted experiments to obtain a hybrid effect of ions and silver nanoparticles as carriers. Instead of synthesizing polarized silver nanoparticles during membrane production, the nanoparticles, which are functional as carriers, eventually formed as a membrane in which silver ions were selected as carriers. The utility of high-mobility ILs as electron acceptors enabled the formation of nanoparticles over time. These nanoparticles were wrapped to polarize the surfaces, thereby enabling them to function as carriers. As a result, the PEO/AgBF_4_/BMIMBF_4_ membrane demonstrated selectivity and permeance values of 15 and 12 GPU, respectively. In contrast, the PEO/AgBF_4_/MOIMBF_4_ membrane exhibited selectivity and permeance values of 17 and 5.3 GPU, respectively [29,30].

In this study, an IL is applied to the copolymer. A poly(ether-block-amide)-5513 (PEBAX-5513)/AgBF_4_/1-butyl-3-methylimidazolium tetrafluoroborate (BMIMBF_4_) composite membrane is prepared. PEBAX-5513 consists of amide (60%) and ether (40%) groups [31]. The amide group helps to stabilize the carrier, whereas the ether group improves the permeance with respect to the flexibility. Therefore, PEBAX-5513 is expected to fabricate a composite membrane with enhanced selectivity when compared to PEO with only ether groups. The prepared composite membrane was analyzed by scanning electron microscopy (SEM), Fourier transform infrared (FTIR) spectroscopy, and thermogravimetric analysis (TGA).

## 2. Materials and Methods

### 2.1. Materials

Silver tetrafluoroborate (AgBF_4_, 98%) and BMIMBF_4_ were purchased from TCI Fine Chemicals (Tokyo, Japan) and Merck (Darmstadt, Germany), respectively. PEBAX-5513 was provided by Arkema Inc. (Colombes, France). All chemicals were used as-received, without further purification.

### 2.2. Preparation of Membrane

The PEBAX-5513/AgBF_4_/BMIMBF_4_ composite membrane was prepared as follows. A PEBAX-5513 solution was prepared using an ethanol–water cosolvent in a weight ratio of 7:3. The AgBF_4_ was dissolved in the solution in a weight ratio of 1:9 compared to the case of PEBAX-5513. The BMIMBF_4_ was added at a molar ratio of 1:0.03 compared to the case of AgBF_4_. The solution was stirred for 20 min. The resulting solution was coated on a polysulfone support (Toray Chemical Korea Inc., Seoul, Korea) using an RK Control Coater (Control Coater RK Print-Coat Instruments Ltd., Litlington, UK). The prepared composite membrane was dried in a vacuum oven at room temperature for ≥ 15 h.

### 2.3. Gas Separation Experiments

The PEBAX-5513/Ag salt/IL composite membrane was subjected to a propane/propylene (50:50 vol%) mixed gas separation experiment. The gas permeance was measured with a bubble flow meter. The permeance was measured in terms of the gas permeation unit (1 GPU = 1 × 10^−6^ cm^3^ (STP)/(s·cm^2^·cm Hg)). The flow rate of the mixed gas was controlled using a mass flow controller (MFC). Gas chromatography (Young Lin 6500 GC system, Young Lin, Gyeonggi, Korea) was used to confirm the propane/propylene selectivity.

### 2.4. Characterization

The cross section of the composite membrane was confirmed using SEM analyses (JEOL JSM-5600LV, JEOL, Tokyo, Japan). The IR measurements were performed using an FTIR spectrometer (VERTEX 70, BRUKER, Billerica, MA, USA); 16 scans were signal averaged with a resolution of 4 cm^−1^. The thermal stability of the membranes was measured using TGA (Universal V4.5 A, TA Instruments, New Castle, DE, USA).

## 3. Results

### 3.1. Separation Performance

**Figure 1:**
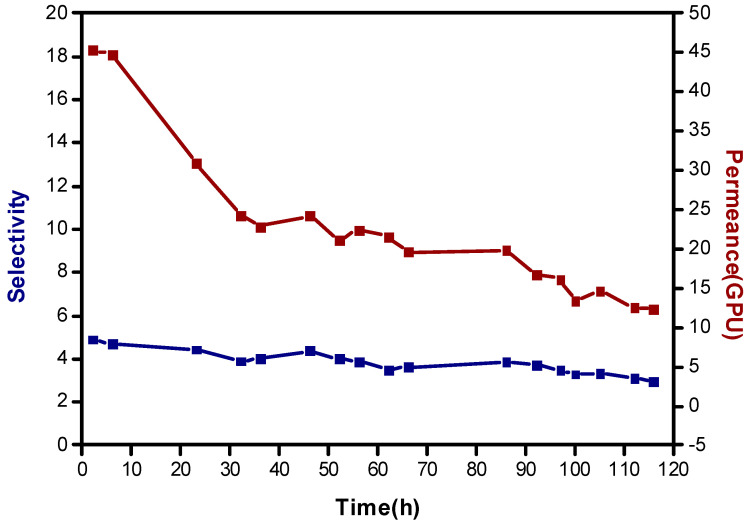
Gas separation performance of the PEBAX-5513/Ag salt/IL composite membrane over time.

Figure 1 depicts the results of the permeation test for the propane/propylene gas mixture. The permeation test was conducted for a duration of ≥100 h. The initial performance of the composite membrane demonstrated a high permeance with selectivity and permeance values of 4.9 and 45.3 GPU, respectively. The final selectivity and permeance of the PEBAX-5513/Ag salt/IL composite membrane at 116 h were 3.0 and 12.3 GPU, respectively. In contrast with the previous PEO/AgBF_4_/BMIMBF_4_ and PEO/AgBF_4_/1-hexyl-3-methyl imidazolium tetrafluoroborate (HMIMBF_4_) membranes, the PEBAX-5513/Ag salt/IL composite membranes were unstable in long-term permeation experiments. The remaining solvent in the membrane was removed, thereby resulting in a decreased flexibility of the polymer and a rapid decrease in the initial permeance. Ag ions were reduced to Ag nanoparticles (AgNPs) over time. These formed AgNPs functioned as a barrier, resulting in a continued decrease in the permeance. Furthermore, the selectivity also decreased because the Ag ions that functioned as olefin carriers became inactive as time progressed. It was confirmed that the formed AgNPs were not properly polarized. Moreover, the ether group was more flexible than the amide group. However, the amide group in PEBAX-5513 occupied a larger proportion (60%), thus making PEBAX-5513 more brittle than PEO, suggesting that BMIMBF_4_ was less adequately dispersed in the PEBAX-5513 matrix than in the PEO matrix.

### 3.2. SEM

**Figure 2:**
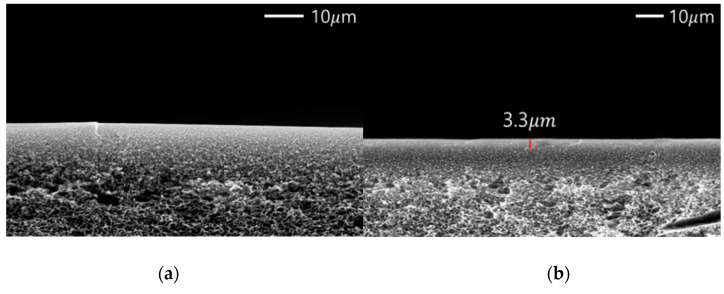
SEM images of the selective layer of (**a**) the neat polymer support and (**b**) the PEBAX-5513/Ag salt/IL membrane coated on the porous polymer support.

The SEM image illustrates the state of the selective layer. Figure 2 shows that the PEBAX-5513/AgBF_4_/BMIMBF_4_ solution was evenly coated on the polysulfone support. The structure of the support resembled a sponge, exhibiting a selection layer of approximately 3.3 μm.

### 3.3. FT-IR

**Figure 3:**
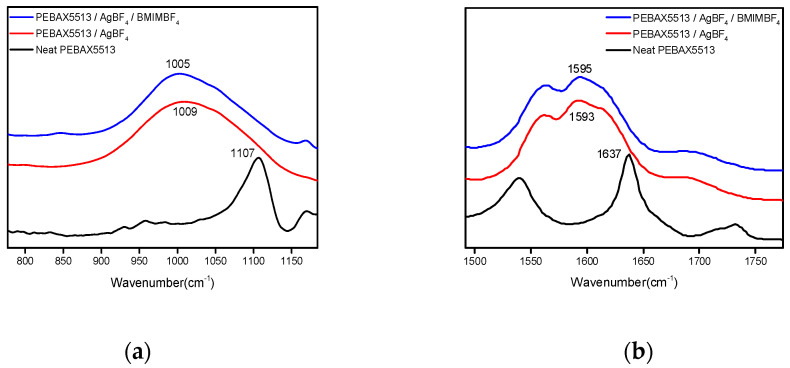
FT-IR spectra of (**a**) ether and (**b**) carbonyl group peaks in neat PEBAX-5513, PEBAX-5513/Ag salt, and PEBAX-5513/Ag salt/IL.

**Figure 4:**
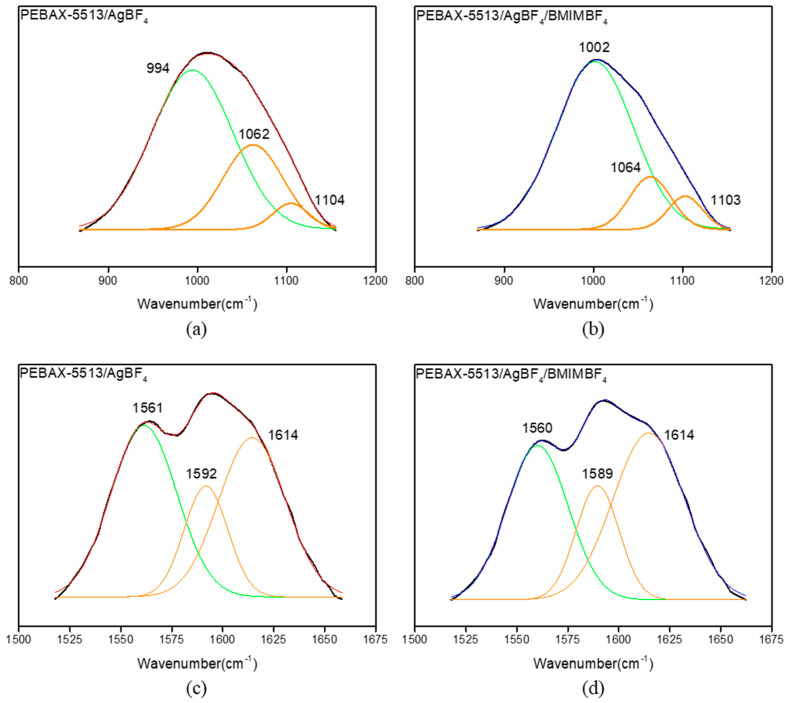
Deconvoluted FT-IR spectra of the ether peak in (**a**) PEBAX-5513/Ag salt and (**b**) PEBAX-5513/Ag salt/IL, and the carbonyl peak in (**c**) PEBAX-5513/Ag salt and (**d**) PEBAX-5513/Ag salt/ILs.

The interactions between PEBAX-5513, AgBF_4_, and BMIMBF_4_ were confirmed using infrared spectroscopy. Figure 3a depicts the change in the C–O stretching bond of PEBAX-5513. The ether peak of neat PEBAX-5513 appeared at 1107 cm^−1^. When AgBF_4_ was added, the ether peak shifted from 1107 to 1009 cm^−1^. The C–O bond was weakened due to the interaction between the Ag ion and the oxygen of the ether group. After BMIMBF_4_ was added, the C–O stretching bond further decreased from 1009 to 1005 cm^−1^. The C–O bond weakened as the BMIM^+^ interacted with the BF_4_^−^ in AgBF_4_. Figure 4 shows the deconvoluted FTIR spectrum that accounted for the degree of peak shift. The peak analysis of ether revealed that the intensity of the 994–1002 cm^−1^ region increased from 68.3% to 79.5% as shown in Table 1. This indicated that BMIMBF_4_ was properly dispersed and BMIM^+^ interacted with BF_4_^−^, resulting in a peak shift after the addition of BMIMBF_4_. Therefore, it was confirmed that BMIMBF_4_ can efficiently penetrate the PE region. The peak deconvolution resulted in a carbonyl group that differed from the peak shift. The area of the 1589–1592 cm^−1^ region increased from 29.2 to 31.2% as shown in Table 2. However, the peak shift and decomposition results were negligible. These results confirmed that the PA region of BMIMBF_4_ was more difficult to penetrate than the flexible PE region. Therefore, long-term stability was not observed because the surface of the particles could not be properly stabilized to induce polarization due to the poor dispersion of the BMIMBF_4_.

**Figure 5:**
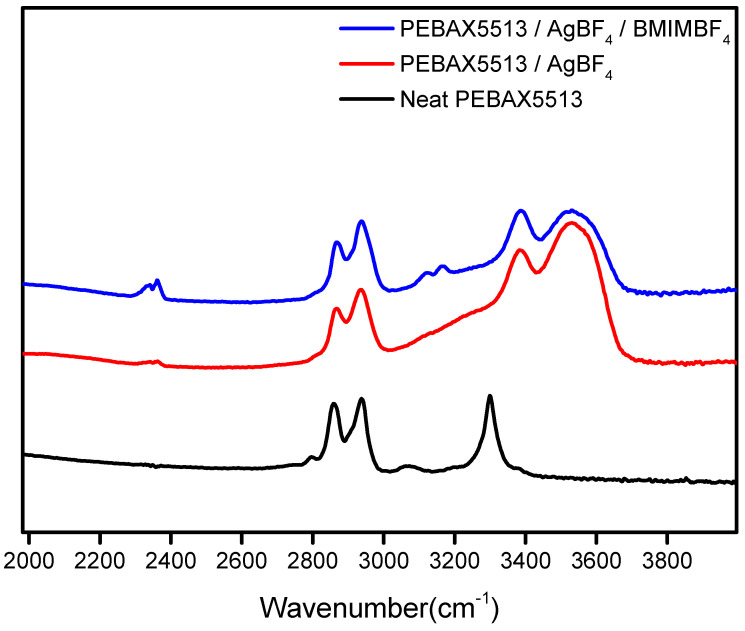
FT-IR spectra of neat PEBAX-5513, PEBAX-5513/Ag salt, and PEBAX-5513/Ag salt/IL.

As shown in Figure 5, the N–H peak of neat PEBAX5513 appeared at 3300 cm^−1^. When AgBF_4_ and BMIMBF_4_ were added, the N–H peak increased to 3390 cm^−1^ and became broadened, confirming that the N–H bond was strengthened. It seemed that some Ag ions interact with the non-covalent electron pair of N, resulting in the weaker C–N bonds and stronger N–H bonds. It was also thought that the intermolecular bond of PEBAX5513 caused the hydrogen bond of N–H bond to be weakened due to Ag ions.

### 3.4. TGA

**Figure 6:**
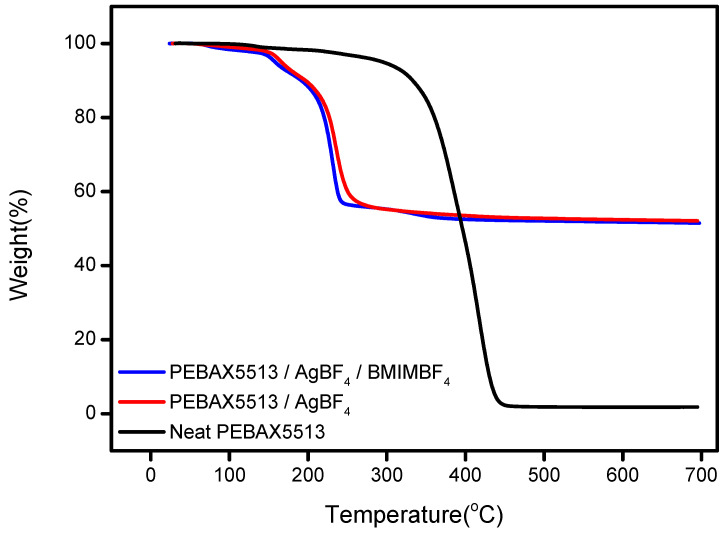
Thermogravimetric analysis (TGA) curves for neat PEBAX-5513, PEBAX-5513/Ag salt, and PEBAX-5513/Ag salt/IL complexes.

The TGA results confirmed a change in the thermal stability of the PEBAX-5513/AgBF_4_/BMIMBF_4_ complex membrane. As shown in Figure 6, the neat PEBAX-5513 experienced a single weight loss between 350 and 450 °C. The PEBAX-5513 polymer containing an amide group exhibited high thermal stability due to intermolecular hydrogen bonding. Following the addition of AgBF_4_, a weight reduction was observed at 200 °C. The reduction in the thermal stability may be attributed to the interaction of Ag ions with both the ether and the amide groups in the polymer, which hindered intermolecular hydrogen bonding. Furthermore, it was suggested that the addition of BMIMBF_4_ disturbed the intermolecular interaction and reduced the thermal stability by the plasticizing effect. However, Figure 6 depicts that the thermal stability was enhanced after 200 °C. These results were attributable to the transient crosslinking effect of Ag ions and the generated Ag nanoparticles.

## 4. Conclusions

**Scheme 1:**
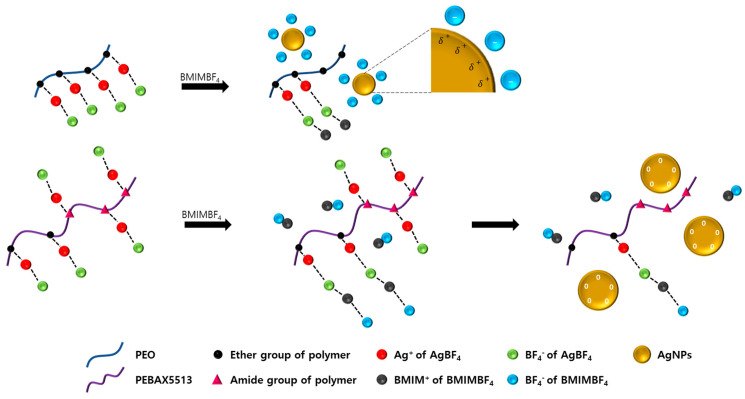
Schematic diagram of the interactions of the PEO/AgBF_4_/BMIMBF_4_ and PEBAX-5513/AgBF_4_/BMIMBF_4_ composite membranes.

In this study, a PEBAX-5513/AgBF_4_/BMIMBF_4_ composite membrane was prepared. The PEBAX-5513 membrane was expected to exhibit a higher stabilizing effect of the amide group on the olefin carriers than the previous PEO/AgBF_4_/BMIMBF_4_ membrane. However, the separation performance of the membrane was not improved. We identified that the BMIMBF_4_ did not easily penetrate the brittle amide region. The FTIR analysis confirmed that the BMIMMBF_4_ exhibited no interaction with the Ag ions in the amide region compared to that in the ether region. Following the formation of nanoparticles, the surface of the NPs could not be surrounded, and the particles were not stabilized as shown in Scheme 1. As a result, the surfaces of the nanoparticles were not polarized and the long-term stability (100 h) was not maintained. Therefore, it is believed that the brittle amide region in the PEBAX-5513 matrix prevented the dispersion of BMIMBF_4_, thereby resulting in poor long-term stability. Consequently, these findings led to the proposal of suitable conditions for the repeating units in polymer matrices when manufacturing and designing membranes with long-term stability for future utilizing ionic liquids.

## Figures and Tables

**Table 1:** Area percentages of each ether peak for the PEBAX-5513, PEBAX-5513/Ag salt, and PEBAX-5513/Ag salt/IL complexes.

Wavenumber (cm^−1^)	PEBAX-5513/AgBF_4_ (%)	PEBAX-5513/AgBF_4_/BMIMBF_4_ (%)
994–1002	68.3	79.5
1062–1064	31.7	20.5
1103–1104		

**Table 2:** Area percentages of each carbonyl peak for the PEBAX-5513, PEBAX-5513/Ag salt, and PEBAX-5513/Ag salt/IL complexes.

Wavenumber (cm^−1^)	PEBAX-5513/AgBF_4_ (%)	PEBAX-5513/AgBF_4_/BMIMBF_4_ (%)
1589–1592	29.2	31.2
1614	70.8	68.8

## References

[1] Sakai M., Sasaki Y., Tomono T., Seshimo M., Matsukata M. (2019). Olefin selective Ag-exchanged X-type zeolite membrane for propylene/propane and ethylene/ethane separation. Acs Appl. Mater. Interfaces.

[2] Bai S., Sridhar S., Khan A. (1998). Metal-ion mediated separation of propylene from propane using PPO membranes. J. Membr. Sci..

[3] Xu L., Rungta M., Brayden M.K., Martinez M.V., Stears B.A., Barbay G.A., Koros W.J. (2012). Olefins-selective asymmetric carbon molecular sieve hollow fiber membranes for hybrid membrane-distillation processes for olefin/paraffin separations. J. Membr. Sci..

[4] Sanchez C.M., Song T., Brennecke J.F., Freeman B.D. (2019). Hydrogen Stable Supported Ionic Liquid Membranes with Silver Carriers: Propylene and Propane Permeability and Solubility. Ind. Eng. Chem. Res..

[5] Jomekian A., Bazooyar B., Poormohammadian S.J., Darvishi P. (2019). A modified non-equilibrium lattice fluid model based on corrected fractional free volume volume of polymers for gas solubility prediction. Korean J. Chem. Eng..

[6] Hamid M.R.A., Jeong H.K. (2018). Recent advances on mixed-matrix membranes for gas separation: Opportunities and engineering challenges. Korean J. Chem. Eng..

[7] Kim J., Do J.Y., Park N., Lee S.J., Hong J., Kang M. (2018). Photoreduction of CO_2_ into CH_4_ using Bi_2_S_3_-TiO_2_ double-layered dense films. Korean J. Chem. Eng..

[8] Raveshiyan S., Hosseini S.S., Karimi-Sabet J. (2020). Intensification of O_2_/N2 separation by novel magnetically aligned carbonyl iron powders/polysulfone magnetic mixed matrix membranes. Chem. Eng. Process..

[9] Sanaeepur H., Mashhadikhan S., Mardassi G., Amooghin A.E., Bruggen B.V., Moghadassi A. (2019). Aminosilane cross-linked poly ether-block-amide PEBAX2533: Characterization and CO_2_ separation properties. Korean J. Chem. Eng..

[10] Dai Y., Li Q., Ruan X., Hou Y., Jiang X., Yan X., He G., Meng F., Wang Z. (2019). Fabrication of defect-free Matrimid^®^ asymmetric membranes and the elevated temperature application for N2/SF6 separation. J. Membr. Sci..

[11] Castro-Dominguez B., Leelachaikul P., Takagaki A., Sugawara T., Kikuchi R., Oyama S.T. (2013). Supported perfluorotributylamine liquid membrane for H2/O_2_ separation. J. Membr. Sci..

[12] Park H.B., Kamcev J., Robeson L.M., Elimelech M., Freeman B.D. (2017). Maximizing the right stuff: The trade-off between membrane permeability and selectivity. Science.

[13] Hunger K., Schmeling N., Jeazet H.B., Janiak C., Staudt C., Kleinermanns K. (2012). Investigation of cross-linked and additive containing polymer materials for membranes with improved performance in pervaporation and gas separation. Membranes.

[14] Beatriz S., Joaquin C., Ignacio G., Miren E.B., Oğuz K., Jürgen C., Freek K., Jorge G. (2015). Metal–organic framework based mixed matrix membranes: A solution for highly efficient CO_2_ capture?. Chem. Soc. Rev..

[15] Roberto C.M., Kumar V.A., Joaquín C. (2020). Ultrathin permselective membranes: The latent way for efficient gas separation. Rsc Adv..

[16] Weston M.H., Colón Y.J., Bae Y., Garibay S.J., Snurr R.Q., Farha O.K., Hupp J.T., Nguyen S.T. (2014). High propylene/propane adsorption selectivity in a copper (catecholate)-decorated porous organic polymer. J. Mater. Chem. A.

[17] Ferraz H., Duarte L., Di Luccio M., Alves T., Habert A., Borges C. (2007). Recent achievements in facilitated transport membranes for separation processes. Braz. J. Chem. Eng..

[18] Marti A.M., Nijem N., Chabal Y.J., Balkus K.J. (2013). Selective detection of olefins using a luminescent silver-functionalized metal organic framework, RPM3. Microporous Mesoporous Mater..

[19] Campos A.C.C., dos Reis R.A., Ortiz A., Gorri D., Ortiz I. (2018). A perspective of solutions for membrane instabilities in olefin/paraffin separations: A review. Ind. Eng. Chem. Res..

[20] Salgado-Gordon H., Valbuena-Moreno G. (2011). Technical and economic evaluation of the separation of light olefins (ethylene and propylene) by using p-complexation with silver salts. Ciencia, Tecnología y Futuro.

[21] Liu Z., Zhang L., Li L., Zhang S. (2019). Separation of olefin/paraffin by electrodialysis. Sep. Purif. Technol..

[22] Merkel T.C., Blanc R., Ciobanu I., Firat B., Suwarlim A., Zeid J. (2013). Silver salt facilitated transport membranes for olefin/paraffin separations: Carrier instability and a novel regeneration method. J. Membr. Sci..

[23] Jeong S., Kang S.W. (2017). Effect of Ag_2_O nanoparticles on long-term stable polymer/AgBF_4_/Al(NO_3_)_3_ complex membranes for olefin/paraffin separation. Chem. Eng. J..

[24] Kang S.W., Kim J.H., Won J., Char K., Kang Y.S. (2003). Enhancement of facilitated olefin transport by amino acid in silver–polymer complex membranes. Chem. Commun..

[25] Dastafkan K., Khajeh M., Ghaffari-Moghaddam M., Bohlooli M. (2015). Silver nanoparticles for separation and preconcentration processes. Trends Anal. Chem..

[26] Han K.I., Kang S.W., Kim J., Kang Y.S. (2011). Effect of ionic liquids on dissociation of copper flake into copper nanoparticles and its application to facilitated olefin transport membranes. J. Membr. Sci..

[27] Ozawa K., Munakata S., Edamoto K., Mase K. (2011). Electron Donor Molecule on the Oxide Surface: Influence of Surface Termination of ZnO on Adsorption of Tetrathiafulvalene. J. Phys. Chem. C.

[28] Lee J.H., Sudhager P., Cho Y., Kang S.W. (2019). Effect of temperature on separation performance in ionic liquid/Ag nanocomposite membranes for olefin/paraffin mixtures. J. Ind. Eng. Chem..

[29] Jeon H., Cho Y., Kang S.W. (2019). Structural Effect of Ionic Liquid on Long-Term Stability in Poly (ethylene oxide)/Ag Ions/Ag Nanoparticles Composite for Olefin Separation. Macromol. Res..

[30] Jeon H., Kang S.W. (2019). Hybrid effect of Ag ions and polarized Ag nanoparticles in poly (ethylene oxide)/AgBF_4_/ionic liquid composites for long-term stable membranes. Polym. Compos..

[31] Jung K.W., Kang S.W. (2019). Effect of functional group ratio in PEBAX copolymer on propylene/propane separation for facilitated olefin transport membranes. Sci. Rep..

